# Exploring modulations in T-cell receptor-mediated T-cell signaling events in systemic circulation and at local disease site of patients with tubercular pleural effusion: An attempt to understand tuberculosis pathogenesis at the local disease site

**DOI:** 10.3389/fmed.2022.983605

**Published:** 2022-12-01

**Authors:** Bhawna Sharma, Diwakar Rathour, Sumbul Uddin, Beenu Joshi, Devendra Singh Chauhan, Santosh Kumar

**Affiliations:** ^1^Department of Immunology, ICMR-National JALMA Institute for Leprosy and Other Mycobacterial Diseases, Agra, UP, India; ^2^Department of Microbiology and Molecular Biology, ICMR-National JALMA Institute for Leprosy and Other Mycobacterial Diseases, Agra, UP, India; ^3^Department of TB and Chest Diseases, Sarojini Naidu Medical College, Agra, UP, India

**Keywords:** tuberculous pleural effusion, T-cell receptor, cytokines, activation, T-cell signaling

## Abstract

**Introduction:**

T cells are crucial for pathogenesis as well as control for tuberculosis (TB). Although much is known about the signaling pathways which are required for the activation of T cells during acute infection but the way these cells respond during persistent of infection still remained elusive. Therefore, it is rationale to understand T cell activation during tuberculous pleural effusion (TPE), which is similar to bacterial persistency system.

**Methods:**

Herein, we will employ T cell receptor (TCR) based approaches for studying events of T cell activation pathways in cells of blood and pleural fluid among patients with TPE. We performed spectrofluorimetric analysis to study effect of M. tuberculosis antigens, ESAT-6 and Ag85A stimulation on intracellular calcium levels, Phosphorylation levels of ZAP-70 (Zeta-chain-associated protein kinase 70), PKC-θ (Protein kinase C theta), Erk1/2 (Extracellular signal-regulated kinase 1 and 2) and p-38 two important members of MAPKs (Mitogen activated Protein kinases) in CD3 and CD28 induced cells of blood and pleural fluid of same patients with TPE by western blotting. Patients with non-TPE were also included as matching disease controls in this study.

**Results:**

We observed significantly higher intracellular calcium levels, Phosphorylation levels of ZAP-70, Erk1/2 and p-38 in CD3 and CD28 induced cells of pleural fluid as compared to the blood cells of same patients with TPE. Alteration in the activation of these events has also been noted after stimulation of ESAT-6 and Ag85A.

**Discussion:**

Present study demonstrated up-regulated activation of TCR mediated T cell signaling events at local disease site (Pleural fluid) as compared to the blood sample of TB pleurisy patients which could be involved in T-cell dysfunctioning during the progression of the disease and also could be responsible for Th 1 dominance at local disease site in patients with TPE.

## Introduction

Globally, an estimated 10 million people fall ill with tuberculosis (TB) worldwide and a total of 1.5 million people die from TB (1). TB remains the leading cause of death globally triggered from a single pathogen, and this TB-related problem is even exacerbated by the human immunodeficiency virus (HIV) infection. *Mycobacterium tuberculosis* (*M. tuberculosis*) elicits an immune response in the host, and the immune cells are activated against the bacteria. Despite activation, the immune system is not capable enough to eliminate bacteria from the body. This goes to prove that *M. tuberculosis* is proficient in altering the immune response for its own survival, leading to disease or latent infection. The molecules and mechanisms utilized to accomplish the survival of bacteria inside the host are not fully understood. The host–pathogen interactions in TB should be analyzed at the disease site because *M. tuberculosis* is predominantly contained in the local tissue lesions. Active TB is characterized by the expansion of *M. tuberculosis*-specific T cells at the site of infection. A very low proportion of *M. tuberculosis*-specific effector T cells are found in the blood compared with the infected tissue, and thus, considerable differences in the cellular immune response and regulatory mechanisms are induced in these diverse compartments. Therefore, it is important to study the immune responses at the local site of infection to improve the understanding of the immunological mechanisms that are involved in the containment and progression of TB. Tuberculous pleural effusion (TPE) is the second most common type of extrapulmonary TB, which is caused by a severe delayed-type hypersensitivity reaction in response to the rupture of a subpleural focus of *M. tuberculosis* infection (2). An accumulation of lymphocytes, in TPE, has been well documented (3–7), which is involved in the pathogenesis of TB pleurisy. The profusion and easy accessibility of pleural fluid mononuclear cells (PFMCs) in the TPE make it a good model to study the *M. tuberculosis*-specific T-cell responses at the local disease site. The signaling pathways triggered by *M. tuberculosis* in human T cells have not yet been studied in a relevant physiological system, hence requiring further investigation. The modulations in T-cell receptor (TCR)-mediated cell signaling mechanism are not completely elucidated to date. It was previously observed that T cells from human TB patients showed a decreased expression of CD3-ζ, a key signaling domain of the TCR/CD3 complex (8). Wang et al. showed that the potent T-cell antigen ESAT-6 can directly suppress interferon-gamma (IFN-γ) production in CD4+ T cells (9). Mahon et al. (10) reported that *M. tuberculosis* cell wall glycolipids directly inhibit polyclonal murine CD4+ T-cell activation by blocking ZAP-70 phosphorylation. Later, they extended their study by reporting on mannose-capped lipoarabinomannan (ManLAM)-induced inhibition of the TCR signaling by interfering with ZAP-70 (zeta-chain-associated protein kinase 70), Lck (lymphocyte-specific protein tyrosine kinase), and LAT (linker for activation of T cells) phosphorylation in antigen-specific murine CD4+ T cells and primary human T cells (11). Palma-Nicholas (12) reported T-cell downmodulation of the mitogen-activated protein kinases–extracellular signal-regulated kinase 1 and 2 (MAPK–ERK1/2) pathway in total spleen cells from naive BALB/c mice by the cell-surface lipid di-O-acyl-trehalose (DAT). Recently, the regulation of interferon-gamma (IFN-γ) production by the ERK and p38 MAPK signaling pathway and through CD150 signaling lymphocytic activation molecule (SLAM) co-stimulation has been suggested to take place in TB (13). The inhibition of IFN-γ production through the p38 MAPK pathway by ESAT-6 has been reported in T cells from healthy individuals (14). We also reported modulations in T-cell signaling events in Jurkat T cells and TB patients (15, 16) in earlier studies. The signaling pathways triggered by *M. tuberculosis* in human T cells have not yet been studied at a local disease site, but there is a previous study that showed that, among pleural fluid lymphocytes, natural killer (NK) cells are a major source of IFN-γ production in a mechanism enhanced by interleukin 12 (IL-12), dependent on calcineurin, p38, and the ERK pathways and these cells can directly recognize *M. tuberculosis* antigens (17). One more study by Chen et al. showed that Toll-like receptor 2 (TLR2) ligand activity was also significantly higher in the tuberculous pleural fluid (TPF) than in the serum. They also observed that *M. tuberculosis* TLR2 ligands, 19-kDa lipoprotein, and live Bacillus Calmette Guerin (BCG) all modulated cytokine production by CD4+ T cells isolated from pleural fluid and activated with anti CD3 and anti CD28 (18). However, there is yet no study on TCR-mediated modulation in T-cell activation in pleural fluid (PF) and peripheral blood (PB) of patients with TB pleurisy. Although very few reports on the alterations of signaling molecules of T-cell activation in patients with TB are available in India. Previously, our study found that the phosphorylation of MAPKs, ERK1/2, and p38 was curtailed by *M. tuberculosis* antigens in patients with TB, whereas, in purified protein derivative (PPD)+ve healthy individuals, only ERK1/2 phosphorylation was inhibited. In addition to this development, we also observed that the binding of transcription factors, such as the nuclear factor of activated T cells (NFAT) and nuclear factor kappa B (NF-κB), was also altered by *M. tuberculosis* antigens (15). The effect of the secretory protein ESAT-6 of *M. tuberculosis* on the modulation of macrophage signaling pathways has been studied (19). Still, there is yet no study to demonstrate whether the mechanisms and molecules were involved in the production of IFN-γ and interleukin 2 (IL-2) (two crucial cytokines for immune responses against TB) by activated T lymphocytes on *M. tuberculosis* stimulation, using any relevant biological model. At the site of the active *M. tuberculosis* infection, as opposed to other forms of TB, PFMCs are readily accessible and provide an opportunity to study the aspects of TB pathogenesis on cells from the local disease site. Thus, it is important to evaluate the immune responses at the local site of infection and in peripheral blood, to improve the understanding of the immunological mechanisms involved in the containment and progression of TB.

## Methodology

### Patient selection

The study protocol was approved by the Institutional Human Ethics Committee of ICMR–National JALMA Institute for Leprosy and Other Mycobacterial Diseases, Agra (India). Informed written consent was obtained from all study subjects. Blood samples and pleural fluid samples were collected from pleurisy patients (*n* = 22) who were aged between 18 and 60 years. Pleural effusions were classified into tuberculous pleural effusion (TPE) (*n* = 15) and non-tuberculous pleural effusion (non-TPE) (*n* = 7) groups. Matching disease controls/patients with non-tuberculous pleural effusion (non-TPE), such as parapneumonic effusion, empyema, and malignancies, were included in the non-TPE group. All patients involved in the study were those who attended the Outpatient Department (OPD) of the Department of *Tuberculosis* and Chest Diseases, Sarojni Naidu Medical College (S.N.M.C), Agra. We also noted their BCG vaccination status and their PPD status, with data shown in Table 1. The effusions were classified as exudates according to at least one of the criteria of Light et al. (20). All patients provided a detailed medical history and underwent a detailed physical examination. The diagnosis of TPE was confirmed on the basis of: medical history, chest X-ray, physical examination, and isolation of *M.tuberculosis* in a positive mycobacterial culture, a smear positive for acid-fast bacilli from the pleural fluid. None of the subjects received anti-tuberculous or steroid therapy at the time of the study. The exclusion criteria included a positive test for human immunodeficiency virus, a pregnant woman, and the presence of concurrent infectious diseases. The diagnosis of tuberculosis was confirmed in all cases by a microscopic examination and culture of *M. tuberculosis* from pleural fluid specimens.

**Table 1:** Demographic data of the study population.

**Characteristics**	**TPE**	**Non-TPE**
Patients (n)=22	15 (68.18%)	7 (31.82%)
**Age**		
Median Range (Lower-Upper)	43 (32.84–47.16)	40 (28.54–52.89)
**Sex**		
Men Women	11 (73.33%) 4 (26.66%)	4 (57.14%) 3 (42.86%)
**BCG**		
Vaccinated Non-vaccinated	11 (73.33%) 4(26.66%)	5 (71.43%) 2 (28.57%)
**PPD status**		
Positive	7 (46.67%)	3 (42.86%)
Negative	8 (53.33%)	4 (57.14%)

TPE, Tuberculous pleural effusion; Non-TPE, Non-tuberculous pleural effusion.

### Thoracentesis and mononuclear cells

Blood samples and pleural fluid samples were collected at the time of therapeutic thoracentesis. Briefly, pleural fluid (PF) (~50 ml) and peripheral blood (10 ml) were obtained from the patients during diagnostic thoracentesis before the initiation of chemotherapy. As much as >50 ml of PF was aspirated under sterile conditions, using 18-gauge needles, collected in heparinized vials and kept in ice during transportation. Blood samples were also collected in heparinized vials and kept at room temperature. Pleural fluid (PF) and blood were obtained simultaneously. Peripheral blood mononuclear cells (PBMCs) and pleural fluid mononuclear cells (PFMCs) were isolated from heparinized blood and pleural fluid (PF) using the standard Ficoll-Hypaque density gradient centrifugation method and suspended in RPMI (Roswell Park Memorial Institute) 1,640 tissue culture medium (Sigma, USA) supplemented with 2 mM L-glutamine, antibiotic–antimycotic solution (Sigma, USA), and 10% heat-inactivated human AB serum (MP Biomedicals, India). Cell cultures were maintained in a humidified 5% CO2 incubator at 37°C. Cell viability ≥95% was determined by the trypan blue exclusion test.

### Chemicals and antigens

Mouse immunoglobulin G (IgG) anti-human pure CD3 (clone UCHT1), ionomycin, goat anti-mouse-IgG (GAM), sodium fluoride (NaF), sodium orthovanadate, anti-protease cocktail and Bradford reagent were purchased from Sigma, USA. Fura-2-acetoxymethyl ester (Fura-2/AM) was procured from Calbiochem, USA. Cell lysis buffer was purchased from Invitrogen, USA. Anti-human CD3 (clone OKT-3) and anti-human CD28 (clone CD28.2) were procured from eBioscience, USA. Phosphorylated ZAP-70, phosphorylated ERK1/2, phosphorylated p38 MAPK, phosphorylated protein kinase C theta (PKC-θ), β-Actin and goat polyclonal IgG anti-mouse peroxidase-conjugated antibodies were procured from Cell Signaling Technology (CST), USA. Enhanced chemiluminescence (ECL) reagents were procured from Millipore, USA. Lyophilized *M. tuberculosis* antigens (ESAT-6 and Ag85A) were procured from the BEI Research Resources Repository funded by the National Institute of Allergy and Infectious Diseases and managed by ATCC, USA. PPD RT-49 (Research Tuberculin) was procured from Statens Serum Institut, Denmark. All antigens were dissolved in filtered phosphate-buffered saline (PBS) at 7.4 pH to make a 1 mg/ml concentration.

### Quantification of transmembrane Ca^2+^ mobilization

Peripheral blood mononuclear cells (PBMCs) and PFMCs (5 × 10^6^/ml) were rested at least for 2 h in a 37°C CO_2_ incubator before stimulation with appropriate doses of *M. tuberculosis* antigens. Cells were stimulated with 5μg/ml Ag85A and 10μg/ml of ESAT-6 for 4 h in a 37°C CO_2_ incubator. After incubation, the cells were washed with PBS, pH 7.4. Cells were incubated with Fura-2/AM at 1 μM for 30 min at 37°C in loading buffer [(in mM): NaCl, 110; KCl, 5.4; NaHCO_3_, 25; MgCl_2_, 0.8; KH_2_PO4, 0.4; HEPES, 20; Na_2_HPO4, 0.33; CaCl_2_, 1.2. pH 7.4]. After loading, cells were washed three times (500×g for 5 min) and remained suspended in an identical buffer. [Ca^2+^]i was measured, as reported elsewhere (21, 22). Fluorescence intensities were measured in ratio mode using a Varian ECLIPSE spectrofluorometer equipped with fast filter accessory (Varian Incorporation, St. Helens, Australia) at 340 and 380 nm (excitation filters) and 510 nm (emission filter). Cells were stirred continuously throughout the experiment. For anti-CD3-stimulated calcium studies: after stabilization of the basal levels of cytosolic calcium, 10 μg/ml of pure anti-CD3 (Clone UCHT1) was added to the cuvette. For the measurement of Fmax, ionomycin 5μM was added to the cuvette and for Fmin, MnCl_2_ 2mM was added to the cuvette.

The intracellular concentrations of free Ca^2+^ [Ca^2+]^i were calculated by using the following equation: [Ca^2+^]i = Kd × (R – Rmin)/(Rmax – R) × (Sf2/Sb2). A value of 224 nM for Kd was added to the calculations. Rmax and Rmin values were obtained by the addition of ionomycin (5 μM) and MnCl_2_ (2 mM), respectively. All experiments were performed at 37°C.

### Treatment and activation of cells

Peripheral blood mononuclear cells (PBMCs) and PFMCs (5 × 10^6^/ml) were stimulated with appropriate doses of *M. tuberculosis* antigens: 5 μg/ml of Ag85A and 10 μg/ml of ESAT-6 for overnight hours in a 37°C CO_2_ incubator, and few cells were left unstimulated. After overnight stimulation with antigens, the cells were activated with plate-bound anti-CD3 and anti-CD28 (clones OKT3 and CD28.2 at 2 μg/ml each procured from eBiosciences). The antibody-coated plates were prepared by coating the wells with goat anti-mouse IgG for 1 h at 37°C; then, the plates were washed and then coated with both anti-CD3 and anti-CD28 also for 1 h at 37°C in a humidified atmosphere of 5% CO_2_. The antigen-stimulated cells were then added to the antibody-coated wells and incubated overnight at 37°C in a humidified atmosphere of 5% CO_2_. Few cells were left unstimulated with antigens and also untreated with CD3 and CD28 antibodies.

### Western blot analysis of MAPK activation

Antigen-stimulated and anti-CD3 and CD28-activated cells were treated with chilled PBS and then lysed with 50 μl of cell lysis buffer (with freshly added 1 mM phenylmethylsulfonyl fluoride (PMSF) and 2 mM anti-protease cocktail). After centrifugation (13,000×g for 5 min), cell lysates were used immediately or stored at −80°C. The protein contents were determined with the Bradford reagent. Denatured proteins (35 μg) were separated by sodium dodecyl sulfate-polyacrylamide (SDS-PAGE) (10%) and transferred to polyvinylidene difluoride (PVDF) membrane. Immunodetection of phosphorylated forms of ZAP-70, PKC-θ, Erk1/2, and p38MAPK was performed using 1:1,000 dilution of phospho-specific antibodies for ZAP-70, PKC-θ, Erk1/2, and p38 MAPK in 5% bovine serum albumin Tris-buffered saline (BSA TBS) for overnight incubation at 4°C. After washing with TBST (TBS with 0.05% Tween 20), the PVDF membranes were treated with peroxidase-conjugated secondary antibodies, and the peroxidase activity was detected with ECL reagents. Equal loading of the proteins was confirmed after stripping the blot and reprobing for total forms of β-Actin. Densitometric analysis of bands was performed using Quantity OneTM software (Bio-Rad, Hercules, USA).

### Statistical analysis

Data were presented as mean ± SEM and comparisons of paired PB and PF samples and different treatments of the same sample were performed using the non-parametric Wilcoxon paired *t*-test. Analysis was done with Prism 5.0 software (GraphPad, La Jolla, USA) and *p*-values <0.05 were considered to indicate statistical significance.

## Results

### TCR triggered intracellular calcium mobilization in pleural fluid and blood samples of TPE patients and matching disease controls and *M. tuberculosis* antigens differentially altered intracellular calcium mobilization

**Figure 1:**
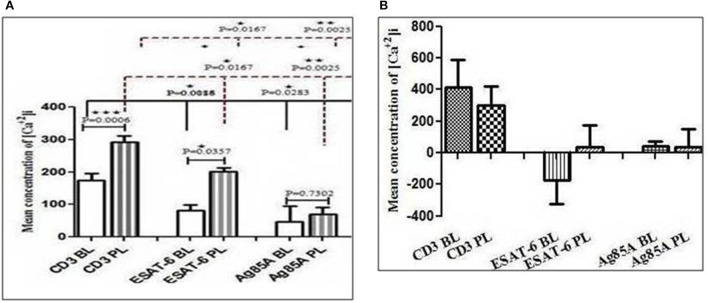
Modification in CD3 (cluster of differentiation 3)-induced free intracellular calcium concentration in blood and pleural fluid after *M. tuberculosis* antigens (ESAT-6 and Ag86A) stimulation: The Fura-2-acetoxymethyl ester (Fura-2/AM)-loaded cells peripheral blood mononuclear cells (PBMCs) and pleural fluid mononuclear cells (PFMCs) were used to study intracellular calcium levels and fluorescence intensities were measured in ratio mode using a Varian ECLIPSE spectrofluorometer, as described in materials and methods section. Bar diagrams show changes in intracellular calcium levels in CD3-treated cells. The effect of *M. tuberculosis* antigens on CD3-stimulated calcium influx is shown in graphs in blood and pleural fluid of patients with tuberculous pleural effusion (TPE) **(A)**, while graph **(B)** is showing the effect of *M. tuberculosis* antigens on blood and pleural fluid of non-TPE patients. The bar is showing the mean ± SEM. *P < 0.05; **P < 0.005; ***P < 0.0005.

To find out the difference between intracellular calcium mobilization in the blood and the pleural fluid and the effect of *M. tuberculosis* antigens on intracellular calcium mobilization, we measured the intracellular calcium concentration with a spectrofluorometer. We assessed the TCR-triggered calcium mobilization by adding anti-CD3 antibodies on cells from the blood and the pleural fluid pretreated with optimum doses of *M. tuberculosis* antigens (Ag85A and ESAT-6). We noticed a significantly higher intracellular level of calcium in cells from the pleural fluid of patients with TPE, as compared to the intracellular calcium levels in their blood after the addition of anti-CD3. We also observed significantly reduced intracellular levels of calcium in ESAT-6 and Ag85A pretreated cells after the addition of anti-CD3 in the blood and the pleural fluid. Interestingly, the reduction was more in blood as compared to the pleural fluid of patients with TPE (Figure 1A). Diminished levels of intracellular calcium were also noted in blood and pleural fluid cells of non-TPE patients, but they were not significant and there was no significant difference between the intracellular calcium levels of blood and pleural fluid of non-TPE patients (Figure 1B).

### TCR-induced ζ-chain-associated 70-KDa tyrosine phosphoprotein (ZAP-70) activation in pleural fluid and blood samples of TPE patients

**Figure 2:**
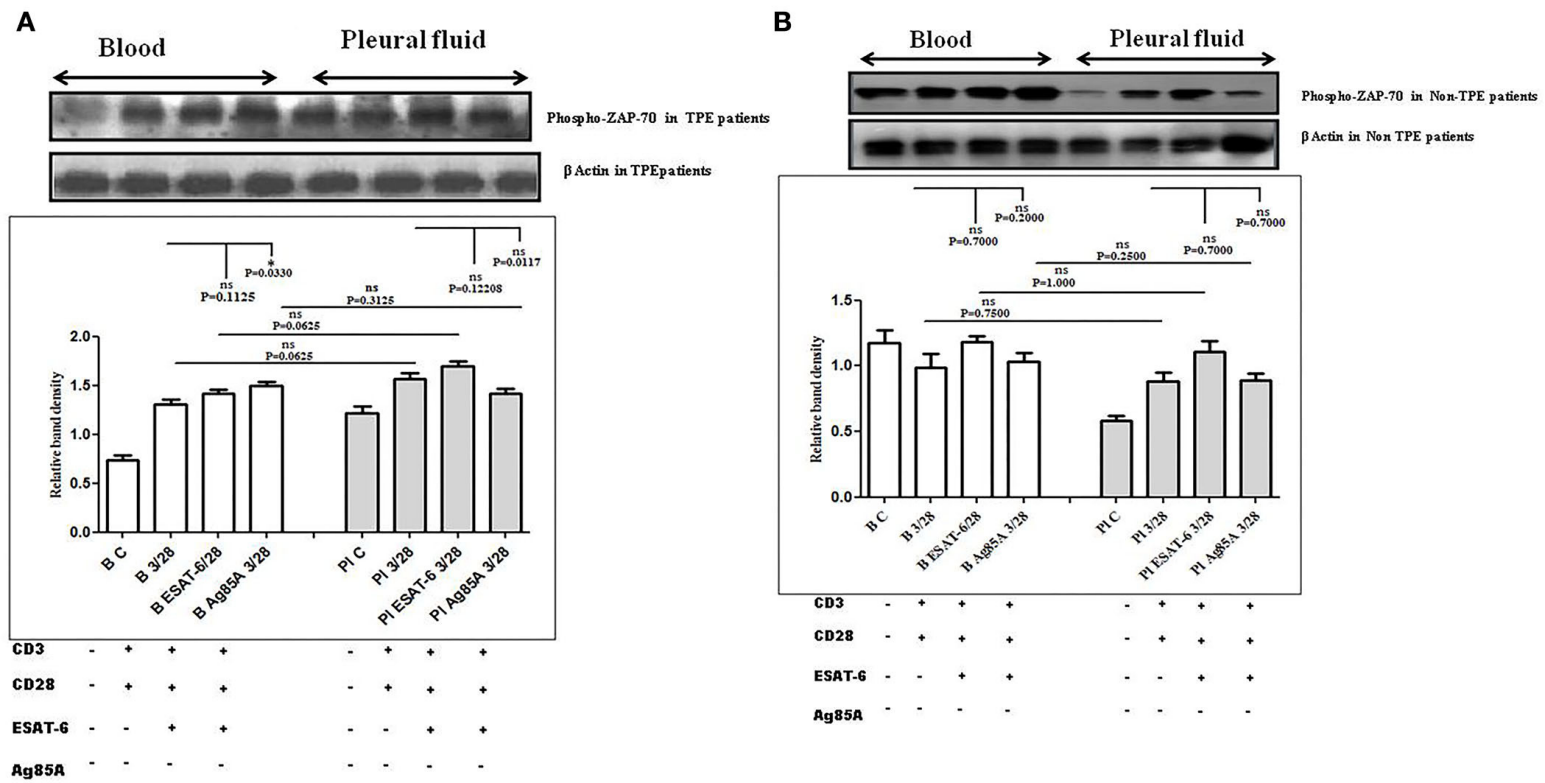
TCR/CD28 (T-cell receptor/cluster of differentiation 28)-induced phosphorylation of zeta-chain-associated protein kinase 70 (ZAP-70) before and after *Mycobacterium tuberculosis* antigen stimulation in blood and pleural fluid of patients with tuberculous pleural effusion (TPE) **(A,B)** non-tuberculosis (non-TB) pleurisy patients. Peripheral blood mononuclear cells (PBMCs) and pleural fluid mononuclear cells (PFMCs) from blood and pleural fluid were activated with CD3 (cluster of differentiation 3) with CD28 antibodies after pretreatment with *M. tuberculosis* antigens, and few cells were left unstimulated and untreated with antibodies as a negative control, and few cells were only activated without any antigen stimulation, and western blotting was done as mentioned in materials and methods. β-Actin antibody was used to conform to equal loading. **(A)** Densitometric analysis of the phosphorylation of ZAP-70 in blood and pleural fluid of patients with TPE. Relative band intensity values are expressed as mean±SEM in bar diagrams. A representative blot of one experiment with phosphorylated Zap-70 and β-Actin is shown, where Lane 1 is showing Control in blood, Lane 2 is showing anti-CD3 + anti-CD28 activated cells in the blood, Lane 3 is showing anti-CD3 + anti-CD28 activated cells of blood with pretreatment with ESAT-6, Lane 4 is showing anti-CD3 + anti-CD28 activated cells of blood pretreated with Ag85A, Lane 5 is showing Control in pleural fluid, Lane 6 is showing anti-CD3 + anti-CD28 activated cells in pleural fluid, Lane 7 is showing anti-CD3 + anti-CD28 activated cells of pleural fluid with pretreatment with ESAT-6, and Lane 8 is showing anti-CD3 + anti-CD28 activated cells of blood pretreated with Ag85A. **(B)** Densitometric analysis of phosphorylation of zeta-chain-associated protein kinase 70 (ZAP-70) in blood and pleural fluid of non-TPE (tuberculous pleural effusion) patients. Relative band intensity values are expressed as mean±SEM in bar diagrams. A representative blot of one experiment with phosphorylated Zap-70 and β-Actin is shown, where Lane 1 is showing Control in blood, Lane 2 is showing anti-CD3 + anti-CD28 activated cells in the blood, Lane 3 is showing anti-CD3 + anti-CD28 activated cells of blood with pretreatment with ESAT-6, Lane 4 is showing anti-CD3 + anti-CD28 activated cells of blood pretreated with Ag85A, Lane 5 is showing Control in pleural fluid, Lane 6 is showing anti-CD3 + anti-CD28 activated cells in pleural fluid, Lane 7 is showing anti-CD3 + anti-CD28 activated cells of pleural fluid with pretreatment with ESAT-6, and Lane 8 is showing anti-CD3 + anti-CD28 activated cells of blood pretreated with Ag85A. Densitometric analysis was done and the ratios of phosphorylated ZAP-70 to β-Actin protein expression were expressed as arbitrary units. Statistical significance was determined using Mann–Whitney *P < 0.05.

To determine whether there is any difference in TCR/CD28-induced ZAP-70 activation in *the* blood and the pleural fluid of patients with TPE and whether *M. tuberculosis* antigens modulate TCR and TCR/CD28-induced ZAP-70 activation, we measured the phosphorylation of ZAP-70 by Western blot. Phosphorylation of ZAP-70 was studied in Ag85A and ESAT-6-stimulated PBMCs and PFMCs of blood and pleural fluid of patients with TPE. We observed significantly higher levels of phosphorylation of ZAP-70 in pleural fluid, as compared to the blood samples of patients with TPE (*n* = 10). After stimulation with *M. tuberculosis* antigens, altered activation of ZAP-70 was observed both in the blood and the pleural fluid (Figure 2A), whereas a significantly increased level of phosphorylated ZAP-70 was observed only in blood after Ag85A stimulation. However, a lower degree of phosphorylation of ZAP-70 was observed in the blood of non-TPE patients (*n* = 5), but not significantly (Figure 2B).

### TCR-induced protein kinase C-theta (PKC-*Θ*) activation in the pleural fluid and the blood samples of patients with TPE and *M. tuberculosis* antigens-induced alterations

**Figure 3:**
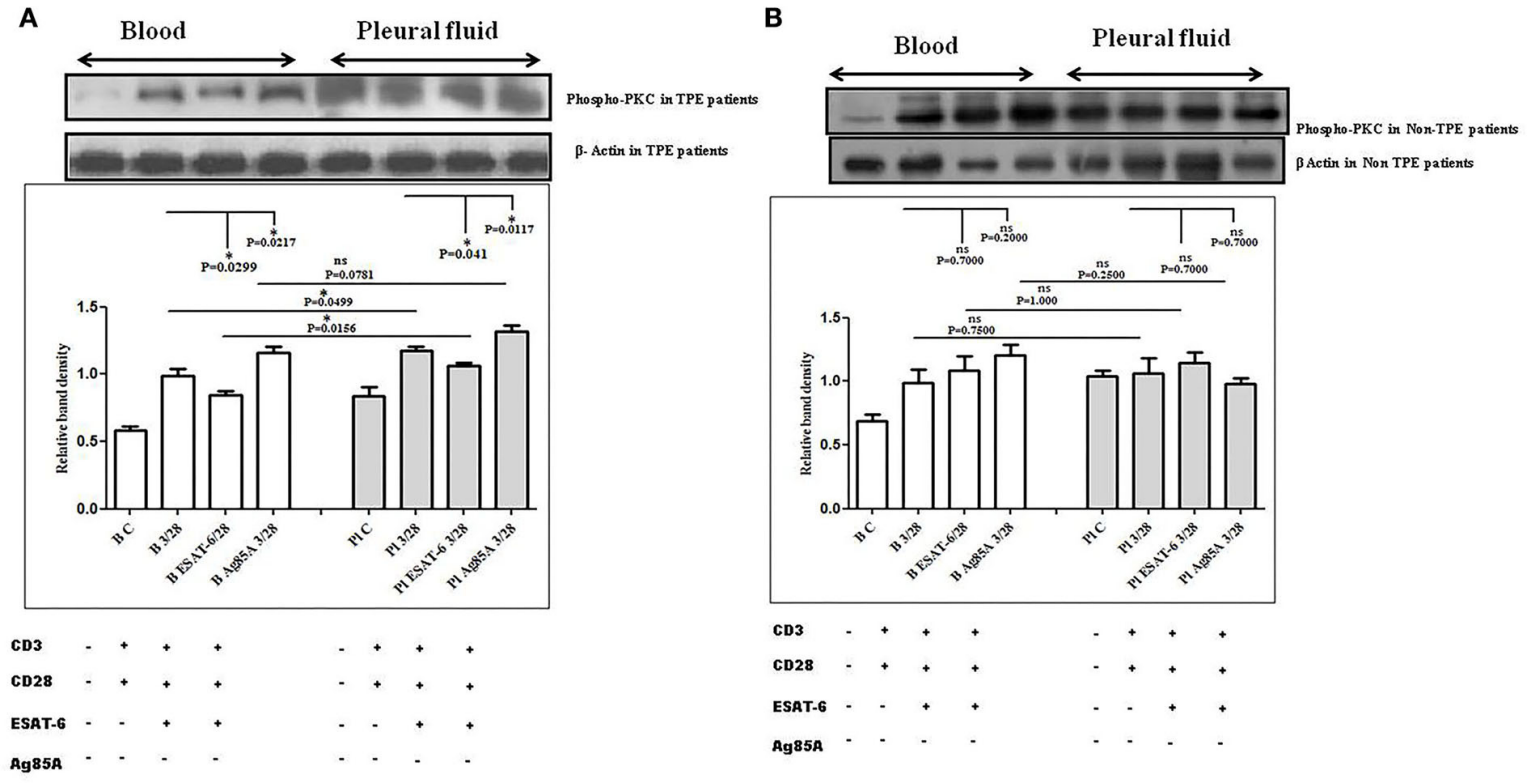
TCR/CD28 (T-cell receptor/cluster of differentiation 28)-induced phosphorylation of protein kinase C theta (PKC-θ) before and after *M. tuberculosis* antigen stimulation in blood and pleural fluid of patients with tuberculous pleural effusion (TPE) **(A,B)** non-TB (non-tuberculosis) pleurisy patients. Peripheral blood mononuclear cells (PBMCs) and pleural fluid mononuclear cells (PFMCs) from blood and pleural fluid were activated with CD3 (cluster of differentiation 3) with CD28 antibodies after pretreatment with *M. tuberculosis* antigens, and few cells were left unstimulated and untreated with antibodies as the negative control, and few cells were only activated without any antigen stimulation, and western blotting was done as mentioned in materials and methods. β-Actin antibody was used to conform to equal loading. **(A)** Densitometric analysis of phosphorylation of PKC-θ in blood and pleural fluid of patients with TPE. Relative band intensity values are expressed as mean±SEM in bar diagrams. A representative blot of one experiment with phosphorylated PKC-θ and β-Actin is shown, where Lane 1 is showing Control in blood, Lane 2 is showing anti-CD3 + anti-CD28 activated cells in the blood, Lane 3 is showing anti-CD3 + anti-CD28 activated cells of blood with pretreatment with ESAT-6, Lane 4 is showing anti-CD3 + anti-CD28 activated cells of blood pretreated with Ag85A, Lane 5 is showing Control in pleural fluid, Lane 6 is showing anti-CD3 + anti-CD28 activated cells in pleural fluid, Lane 7 is showing anti-CD3 + anti-CD28 activated cells of pleural fluid with pretreatment with ESAT-6, and Lane 8 is showing anti-CD3 + anti-CD28 activated cells of blood pretreated with Ag85A. **(B)** Densitometric analysis of phosphorylation of PKC-θ in blood and pleural fluid of non-TPE patients. Relative band intensity values are expressed as mean±SEM in bar diagrams. A representative blot of one experiment with phosphorylated PKC-θ and β-Actin is shown, where Lane 1 is showing Control in blood, Lane 2 is showing anti-CD3 + anti-CD28 activated cells in the blood, Lane 3 is showing anti-CD3 + anti-CD28 activated cells of blood with pretreatment with ESAT-6, Lane 4 is showing anti-CD3 + anti-CD28 activated cells of blood pretreated with Ag85A, Lane 5 is showing Control in pleural fluid, Lane 6 is showing anti-CD3 + anti-CD28 activated cells in pleural fluid, Lane 7 is showing anti-CD3 + anti-CD28 activated cells of pleural fluid with pretreatment with ESAT-6, and Lane 8 is showing anti-CD3 + anti-CD28 activated cells of blood pretreated with Ag85A. Densitometric analysis was done and the ratios of phosphorylated PKC-θ to β-Actin protein expression were expressed as arbitrary units. Statistical significance was determined using Mann–Whitney *P < 0.05.

To determine the differences in TCR/CD28-induced PKC-θ activation in the blood and the pleural fluid of patients with TPE and to study the effect of *M. tuberculosis* on TCR/CD28-induced PKC-θ activation, we measured the phosphorylation of PKC-θ by Western blot. We observed significantly higher levels of phosphorylated PKC θ in pleural fluid, as compared to the blood samples of TPE patients (*n* = 10). After stimulation with *M. tuberculosis* antigens, a significantly altered activation was observed both in the blood and the pleural fluid. ESAT-6 significantly reduced the phosphorylation of PKC θ and Ag85A significantly increased phosphorylated PKC θ levels both in the blood and the pleural fluid. The activation of PKC θ was more in pleural fluid as compared to the blood before and after stimulation with *M. tuberculosis* antigen stimulation (Figure 3A). A higher activation of PKC θ was also observed in non-TPE patients (*n* = 5) but not significantly (Figure 3B).

### Differences in TCR-induced mitogen-activated protein kinases (MAPKs) activation in pleural fluid and blood samples of patients with TPE and *M. tuberculosis* antigens-induced alterations

**Figure 4:**
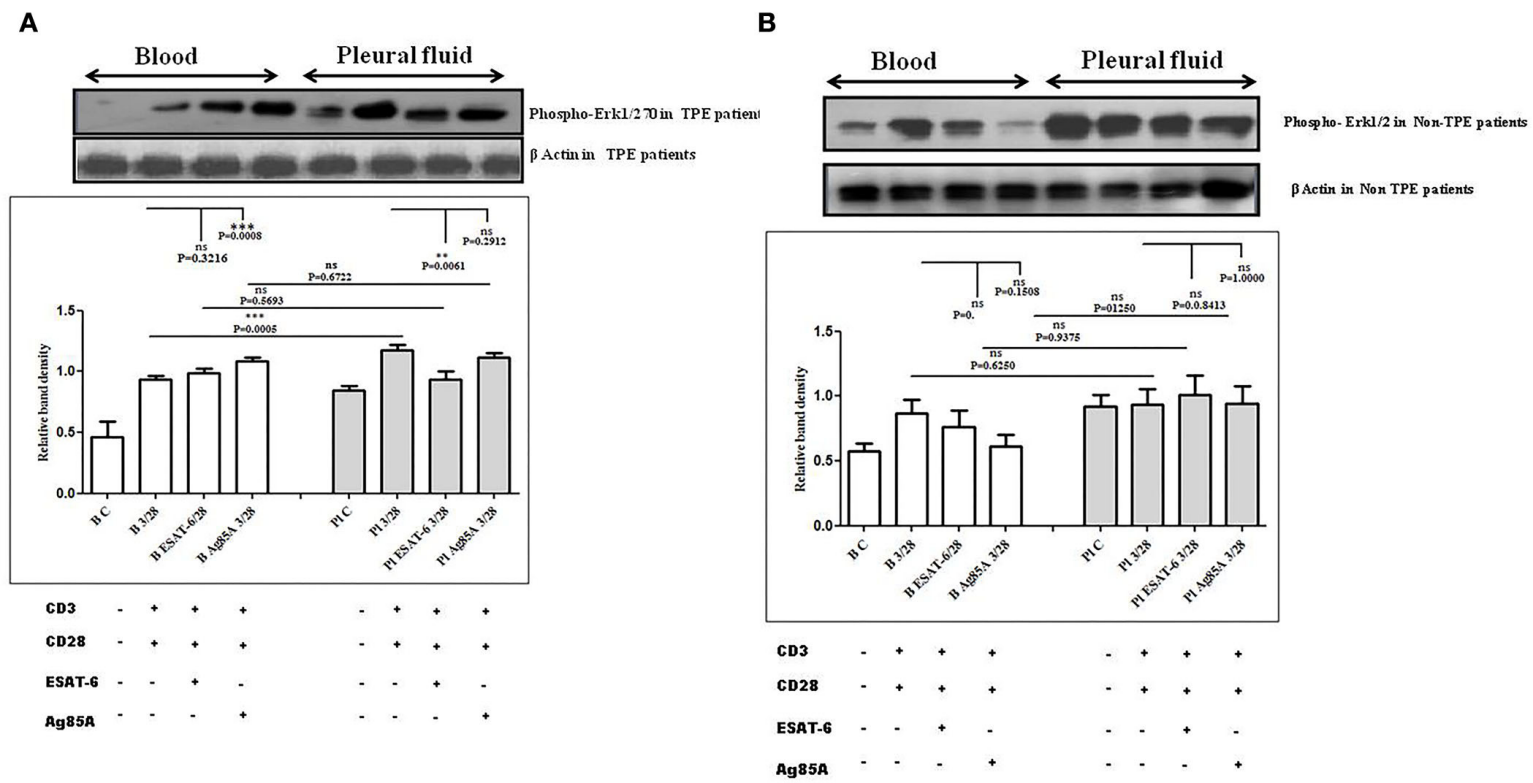
TCR/CD28 (T-cell receptor/cluster of differentiation 28)-induced phosphorylation of Erk1/2 before and after *M. tuberculosis* antigen stimulation in blood and pleural fluid of patients with tuberculous pleural effusion (TPE) **(A,B)** non-TPE patients. Peripheral blood mononuclear cells (PBMCs) and pleural fluid mononuclear cells (PFMCs) from blood and pleural fluid were activated with CD3 with CD28 antibodies after pretreatment with *M. tuberculosis* antigens, few cells were left unstimulated and untreated with antibodies as the negative control, and few cells were only activated without any antigen stimulation, and western blotting was done as mentioned in materials and methods. β-Actin antibody was used to conform to equal loading. **(A)** Densitometric analysis of phosphorylation of Erk1/2 in blood and pleural fluid of patients with tuberculosis (TB) pleurisy. Relative band intensity values are expressed as mean±SEM in bar diagrams. A representative blot of one experiment with phosphorylated Erk1/2 and β-Actin is shown, where Lane 1 is showing Control in blood, Lane 2 is showing anti-CD3 + anti-CD28 activated cells in the blood, Lane 3 is showing anti-CD3 + anti-CD28 activated cells of blood with pretreatment with ESAT-6, Lane 4 is showing anti-CD3 + anti-CD28 activated cells of blood pretreated with Ag85A, Lane 5 is showing Control in pleural fluid, Lane 6 is showing anti-CD3 + anti-CD28 activated cells in pleural fluid, Lane 7 is showing anti-CD3 + anti-CD28 activated cells of pleural fluid with pretreatment with ESAT-6, and Lane 8 is showing anti-CD3 + anti-CD28 activated cells of blood pretreated with Ag85A. **(B)** Densitometric analysis of the phosphorylation of Erk1/2 in blood and pleural fluid of non-TB (non-tuberculosis) patients with TB pleurisy. Relative band intensity values are expressed as mean±SEM in bar diagrams. A representative blot of one experiment with phosphorylated Erk1/2 and β-Actin is shown, where Lane 1 is showing Control in blood, Lane 2 is showing anti-CD3 + anti-CD28 activated cells in the blood, Lane 3 is showing anti-CD3 + anti-CD28 activated cells of blood with pretreatment with ESAT-6, Lane 4 is showing anti-CD3 + anti-CD28 activated cells of blood pretreated with Ag85A, Lane 5 is showing Control in pleural fluid, Lane 6 is showing anti-CD3 + anti-CD28 activated cells in pleural fluid, Lane 7 is showing anti-CD3 + anti-CD28 activated cells of pleural fluid with pretreatment with ESAT-6, and Lane 8 is showing anti-CD3 + anti-CD28 activated cells of blood pretreated with Ag85A. Densitometric analysis was done and the ratios of phosphorylated Erk1/2 to β-Actin protein expression were expressed as arbitrary units. Statistical significance was determined using Mann–Whitney ** *P* < 0.005; *** *P* < 0.005.

**Figure 5:**
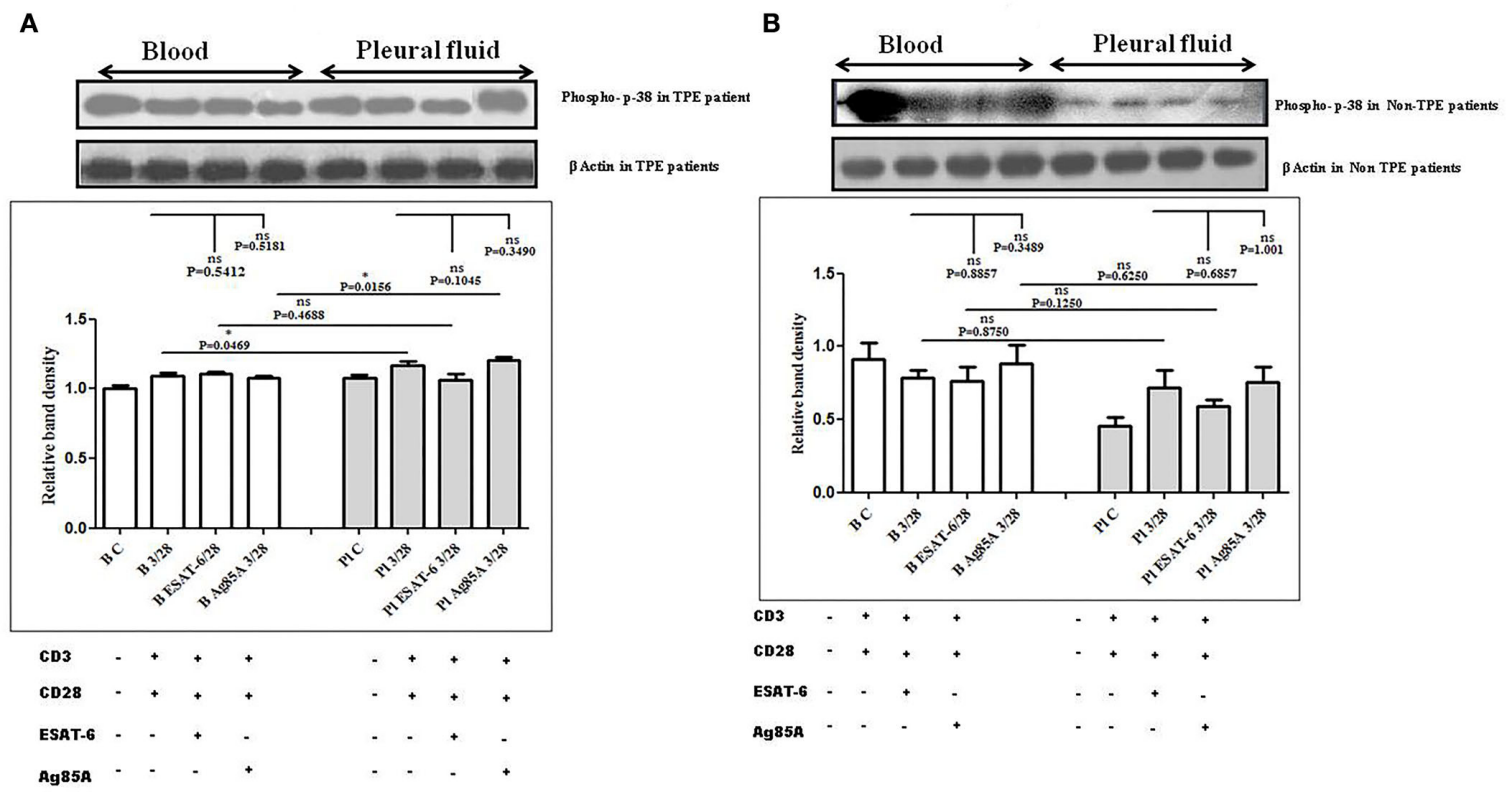
TCR/CD28 (T-cell receptor/cluster of differentiation 28)-induced phosphorylation of p38 before and after *M. tuberculosis* antigen stimulation in blood and pleural fluid of patients with tuberculous pleural effusion (TPE) **(A,B)** non-TPE patients. Peripheral blood mononuclear cells (PBMCs) and pleural fluid mononuclear cells (PFMCs) from blood and pleural fluid were activated with CD3 (cluster of differentiation 3) with CD28 antibodies after pretreatment with *M. tuberculosis* antigens, and few cells were left unstimulated and untreated with antibodies as a negative control, and few cells were only activated without any antigen stimulation, and western blotting was done as mentioned in materials and methods. β-Actin antibody was used to conform to equal loading. **(A)** Densitometric analysis of phosphorylation of p38 in blood and pleural fluid of patients with TPE. Relative band intensity values are expressed as mean±SEM in bar diagrams. A representative blot of one experiment with phosphorylated p38 and β-Actin is shown, where Lane 1 is showing Control in the blood, Lane 2 is showing anti-CD3 + anti-CD28 activated cells in the blood, Lane 3 is showing anti-CD3 + anti-CD28 activated cells of blood with pretreatment with ESAT-6, Lane 4 is showing anti-CD3 + anti-CD28 activated cells of blood pretreated with Ag85A, Lane 5 is showing Control in pleural fluid, Lane 6 is showing anti-CD3 + anti-CD28 activated cells in pleural fluid, Lane 7 is showing anti-CD3 + anti-CD28 activated cells of pleural fluid with pretreatment with ESAT-6, and Lane 8 is showing anti-CD3 + anti-CD28 activated cells of blood pretreated with Ag85A. **(B)** Densitometric analysis of phosphorylation of p38 in blood and pleural fluid of non-TPE patients. Relative band intensity values are expressed as mean±SEM in bar diagrams. A representative blot of one experiment with phosphorylated p38 and β-Actin is shown, where Lane 1 is showing Control in the blood, Lane 2 is showing anti-CD3 + anti-CD28 activated cells in the blood, Lane 3 is showing anti-CD3 + anti-CD28 activated cells of blood with pretreatment with ESAT-6, Lane 4 is showing anti-CD3 + anti-CD28 activated cells of blood pretreated with Ag85A, Lane 5 is showing Control in pleural fluid, Lane 6 is showing anti-CD3 + anti-CD28 activated cells in pleural fluid, Lane 7 is showing anti-CD3 + anti-CD28 activated cells of pleural fluid with pretreatment with ESAT-6, and Lane 8 is showing anti-CD3 + anti-CD28 activated cells of blood pretreated with Ag85A. Densitometric analysis was done and the ratios of phosphorylated p38 to β-Actin protein expression were expressed as arbitrary units. Statistical significance was determined using Mann–Whitney *P < 0.05.

To determine the differences in TCR/CD28-induced MAPK activation in blood and pleural fluid of patients with TPE and to study the effect of *M. tuberculosis* on TCR/CD28-induced MAPKs activation, we measured the phosphorylation of Erk $\frac{1}{2}$ and p38 by Western blot. The phosphorylation of MAPKs was studied in Ag85A and ESAT-6-stimulated PBMCs and PFMCs. We observed significantly higher levels of phosphorylation of Erk $\frac{1}{2}$ in pleural fluid as compared to the blood of patients with TPE (*n* = 12), and after stimulation with *M. tuberculosis* antigens, altered activation of Erk $\frac{1}{2}$ was observed both in th4 blood and pleural fluid. Increased phosphorylation of Erk $\frac{1}{2}$ was observed in blood after *M. tuberculosis* antigen stimulation Ag85A significantly increased, while it was not significant after ESAT-6 stimulation in blood. On the other hand, in pleural fluid, significantly increased phosphorylation was observed after ESAT-6 stimulation but not significantly with Ag85A stimulation (Figure 4A). Altered levels of phosphorylated Erk$\frac{1}{2}$ were also observed in the blood and the pleural fluid of non-TPE patients (*n* = 5), but not significantly (Figure 4B). We observed significantly increased phosphorylation of p38 in pleural fluid and blood of patients with TPE (*n* = 12), and after stimulation with *M. tuberculosis* antigens, altered activation was observed both in the blood and the pleural fluid, but not significantly. p38 phosphorylation was more significantly increased in the pleural fluid than in the blood after stimulation with Ag85A; on the other hand, decreased phosphorylation of p38 was observed after ESAT-6 stimulation but it was not significant (Figure 5A). We also observed reduced p38 phosphorylation in the pleural fluid of non-TPE patients as compared to blood (*n* = 5) but not significantly (Figure 5B).

## Discussion

TB pathogenesis is driven by a complex interplay between the host immune system and the survival strategies of *M. tuberculosis*. The inflammatory response to *M. tuberculosis* infection is tightly regulated by both the host and the pathogen and the protection against TB is based on cell-mediated immune responses. A consistent feature in TB patients has been the *in vitro* dysfunction of circulating T lymphocytes and mononuclear cells, especially at chronic stages of the disease (23). The real progress requires more detailed knowledge of the host immune responses. Mycobacterial species are well adapted to the hostile environment of phagocytic cells, and they use several strategies for survival within the host cells that are not seen in other bacteria. Our understanding of the mechanisms of interaction between mycobacteria and host cells, and of the consequent changes that are induced by mycobacteria in the host signaling machinery, is still incomplete.

T-cell activation and proliferation require the binding of the TCR complex and CD4 or CD8 (cluster of differentiation 8) receptor to specific major histocompatibility complex (MHC) bound antigens. Co-stimulatory signals delivered by the interaction of T-cell membrane molecules and antigen-presenting cells (APCs) also play an important role in the complete activation of a T cell. After the activation, the intracellular calcium level rises and cytoplasmic kinases also get activated, which further leads to the translocation of nuclear transcription factors. The Lck binding acts as a docking site for ZAP-70, which converts the TCR to an active protein tyrosine kinase that is able to phosphorylate, leading to the generation of downstream signals, and the resting T cell enters the cell cycle, inducing various genes to produce cytokines and further go for differentiation and apoptosis (24). Any impairment in the T-cell signaling cascade leads to defects in cellular responses. There are many stages in the T-cell activation pathway, which have not been thoroughly explored in the case of TB. The effect of *M. tuberculosis* infection leads to a modulation in the T- cell activation pathway, which should be investigated to understand the pathogenesis of *M. tuberculosis* completely.

We proposed that, to explore pathogenesis of TB with the aim to develop new strategies for prevention and treatment, host–pathogen interactions should be studied at the local site of *M. tuberculosis* infection. TPE is of particular immunological interest and the study of T-cell activation mechanism in TPE provides an opportunity to evaluate a component of the human immune response at the infection site that is probably involved in mediating the containment of *M. tuberculosis in vivo*. The signaling pathways triggered by *M. tuberculosis* in human T cells have not yet been studied in a relevant physiological system. The presence of mycobacterial antigens in the pleural space elicits an intense cellular immune response, resulting in lymphocyte-predominant exudative effusions (3, 25) making this biological sample a physiologically relevant model of human tuberculosis infection. We used the pleural fluid of the patient with TPE as a sample from the local disease site and a blood sample as the sample from systemic circulation.

The signaling pathways triggered by *M. tuberculosis* in human T cells have not yet been studied in a relevant physiological system like TPE. One previous study showed that, among pleural fluid lymphocytes, natural killer (NK) cells are a major source of IFN-γ production in a mechanism enhanced by IL-12, dependent on calcineurin, p38, and Erk1/2 pathways, and these cells can directly recognize *M. tuberculosis* antigens (17). One more study by Chen et al. showed that TLR2 ligand activity was also significantly higher in the tuberculous pleural fluid than in the serum. They also observed that *M. tuberculosis* TLR2 ligands, 19-kDa lipoprotein, and live BCG all modulated cytokine production by CD4+ T cells isolated from pleural fluid and activated with anti-CD3 and anti-CD28 (18). However, no study is available for TCR-mediated modulation in T-cell activation in the pleural fluid and the peripheral blood of patients with TPE.

**Figure 6:**
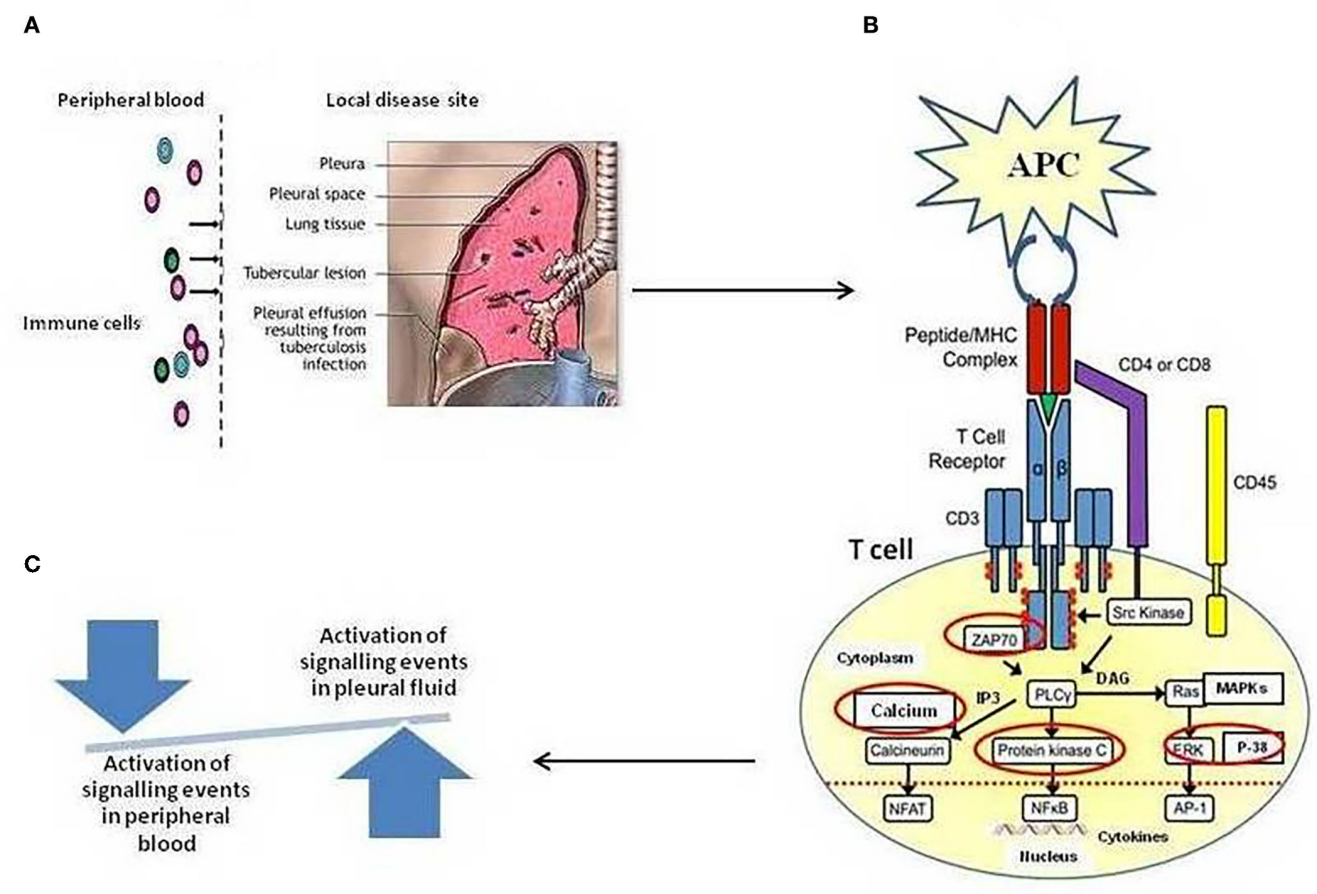
**(A)** Schematic illustration of Compartmentalization of different immune cell subsets at the local site/tuberculous pleural effusion (TPE) of *Mycobacterium tuberculosis* infection. High frequencies of *in vivo*-activated *M. tuberculosis*-specific T cells at local disease site as compared to peripheral blood, showing a snapshot of a specific temporal window of tuberculosis disease **(B)**. Regulation of T-cell receptor (TCR)-mediated T-cell activation mechanism. The figure depicts the activation of various enzymes and adaptor molecules upon engagement of TCR with the major histocompatibility complex (MHC) antigenic peptide complex. The red-circled molecules are the molecules whose activation is being studied. **(C)** A shift in the local immune response not only tips the balance toward suboptimal immunity and impaired control of tuberculosis disease but also results in excessive cellular immunity and tissue destruction.

Herein, we employed TCR-based approaches for studying events pertaining to T-cell activation pathways in cells of blood and tuberculous pleural fluid among patients with TPE. In the present study, we hypothesized that the T cells in tuberculous pleural effusion can directly recognize *M. tuberculosis* antigens and can be activated more rapidly in comparison to the cells from peripheral blood in a mechanism dependent on TCR-mediated activation pathway involving TCR-induced intracellular Ca^+2^ mobilization, ZAP-70 activation, PKC-θ activation, and MAPKs' activation that regulates the transcription of IFN-γ and IL-2. As shown in Figure 6, a pictorial representation of the hypothesis. Host defense against TB infection involves T-lymphocyte-mediated cellular immune responses, and to understand TB pathogenesis, signaling pathways induced by mycobacteria have long been a subject of interest.

The intracellular Ca^2+^ signaling is critical to T-cell activation as a means of rapidly activating and integrating numerous signaling pathways to generate widespread changes in T-cell gene expression and function. Ca^2+^ is a second messenger that functions by binding to and altering the function of key proteins leading to pleiotropic changes in cell function. Binding of antigen to the TCR triggers the mobilization of Ca^2+^ and is needed for effective T-cell activation, anergy, gene expression, motility, synapse formation, cytotoxicity, development, and differentiation of T cells (26). ZAP-70 is a cytoplasmic protein tyrosine kinase that plays a critical role in the events involved in initiating T-cell responses by the antigen receptor. Protein kinase C theta (PKC-θ) is a key kinase in mediating TCR signals. PKC-θ activated by TCR engagement translocates to immunological synapses (ISs) and regulates the activation of transcriptional factors NF-κB, AP-1, and NFAT. These transcription factors then activate target genes such as IL-2 and IFN-γ. Activation of mitogen-activated protein kinases (MAPKs) further downstream to calcium/PKC is an important event for both cytokine production and cell activation. Serine/threonine protein kinases-MAPKs comprise a family of protein kinases, including extracellular signal-regulated kinases 1 and 2 (Erk1/2), p38 MAPK, and c-Jun N-terminal kinase (JNK), which have been implicated as important cellular signaling molecules activated by mycobacteria (26). CD4+ T cells play a central role in the containment of *M. tuberculosis* infection by secreting IFN-γ and IL-2 (27). The modulations in the TCR-mediated cell signaling mechanism are not completely elucidated to date. Although very few reports of alterations in signaling molecules are available so far, this study used Jurkat T cells, peripheral blood either from humans or from mice (10–12, 15, 16).

In the present study, significantly higher CD3-induced Ca^2+^ levels were observed in pleural fluid as compared to blood and we also noted modulation in the Ca^2+^ level after *M. tuberculosis* antigen stimulation in blood and pleural fluid of patients with TPE. In a previous study, we observed that ESAT-6 significantly downregulated intracellular Ca^2+^ in the blood of pulmonary TB patients (15). We also noted the same observation in this study and our finding showed that ESAT-6 reduced intracellular Ca^2+^ in blood and pleural fluid of TPE patients. Direct inhibitory mechanisms of T-cell activation has until now not been extensively studied. It was previously observed that T cells from human TB patients had decreased expression of CD3-ζ, a key signaling domain of the TCR/CD3 complex (8). Mahon et al. found that *M. tuberculosis* bacilli directly inhibited CD4+ T-cell activation. Mahon et al. (10) reported that *M. tuberculosis* cell wall glycolipids directly inhibit polyclonal murine CD4+ T-cell activation by blocking ZAP-70 phosphorylation and later they extended their study by reporting that ManLAM induced inhibition of TCR signaling by interfering with ZAP-70, Lck, and LAT phosphorylation in antigen-specific murine CD4+ T cells and primary human T cells (11). In the present study, we studied ZAP-70 activation in blood and pleural fluid of anti-CD3 and CD28-activated cells after being pretreated with *M. tuberculosis* antigen. We observed the increased activation of ZAP-70 in pleural fluid, as compared to blood in TPE patients. We observed significantly higher levels of phosphorylation of ZAP-70 in pleural fluid, as compared to blood samples of TPE patients. After stimulation with *M. tuberculosis* antigens, altered activation was observed both in blood and pleural fluid, whereas the significantly increased level of phosphorylated ZAP-70 was observed only in the blood after Ag85A stimulation. On the other hand, lesser phosphorylation of ZAP-70 was observed in non-TPE patients but not significantly. The ZAP-70 activation regulates further activation of other downstream signaling molecules, so we further studied the activation of PKC-θ. PKC-θ is the first PKC family member described to be recruited to the immunological synapse (IS) (28). It plays an integral role in activating a range of signaling cascades that ultimately result in a transcriptional network in T cells. PKC includes a large family of homologous serine/threonine protein kinases that are widely conserved in eukaryotes (29). We observed significantly higher levels of phosphorylated PKC θ in pleural fluid, as compared to blood samples of TPE patients. After stimulation with *M. tuberculosis* antigens, significantly altered activation was observed in blood and pleural fluid both. Higher activation of PKC θ was also observed in non-TPE patients but not significantly. We also observed the activation of MAPKs and higher activation of Erk1/2 and p38 in pleural fluid as compared to blood samples and alteration in the activation of these MAK molecules was also noted after stimulation with ESAT-6 and Ag85A. We did not find any significant change in the activation of MAPKs in blood and pleural fluid samples of non-TPE patients. The earlier studies of the T-cell activation mechanism in TB have investigated various events of T-cell signaling. Wang et al. showed that the potent T-cell antigen ESAT-6 can directly suppress IFN-γ production in CD4+ T cells (9), Palma-Nicholas (12) reported T-cell downmodulation of the MAPK–ERK1/2 pathway in total spleen cells from naive BALB/c mice by the cell-surface lipid di-O-acyl-trehalose (DAT). The regulation of IFN-γ production by the ERK and p38 MAPK signaling pathway, through SLAM co-stimulation, had been studied in TB (13). Inhibition of IFN-γ production through the p38 MAPK pathway by ESAT-6 was reported in T cells from healthy individuals (14). The effect of the secretory protein ESAT-6 of *M. tuberculosis* on the modulation of macrophage signaling pathways was also studied earlier (19). Our previous study showed that the phosphorylation of MAPKs–Erk1/2 and p38 was curtailed by *M. tuberculosis* antigens in patients with TB, whereas in PPD+ve healthy individuals only Erk1/2 phosphorylation was inhibited, the inhibitory effect of secretory antigens of *M. tuberculosis* on the modulation of T-cell signaling pathways was also observed in this study (15).

*M. tuberculosis* is capable of establishing infection in the host by altering the different cell machineries. It modulates the cell processes according to itself for the survival inside the host. The modulation in T-cell signaling is not explored enough, but *M. tuberculosis* is strongly thought of to cause changes in the signal transduction pathway. This study explored the mechanisms in *M. tuberculosis* infection, which are used to destabilize the host's T-cell response, besides what are already established. Although previous studies of cell signaling pathways in TB have contributed to marked advances in our knowledge about their role in host protective immune responses, a number of critical questions are still unstated. Research into the development of TB vaccines and immunodiagnostics has focused on the proteins released by *M. tuberculosis*, because these antigens are thought to induce protective cell-mediated immunity and immune responses of diagnostic value. Ag85A and ESAT-6 are widely studied for their potential to trigger effective host immune responses against TB, but our knowledge regarding their role in the T-cell signaling mechanisms underlying proinflammatory cytokine secretion by T cells is not well established. Presently, follow-up studies are also needed to determine whether such alterations in the activation mechanism revert after successful treatment and also whether these can be modulated by immunotherapy. It is also of interest to know the variations in different forms of TB and to determine the relationship with the activation of different cytokines. These observations of molecular and functional characteristics in TB may provide new tools to analyze and monitor patients, to reveal how these characteristics affect the development of immune dysfunction, and to study new pathways to block suppressor mechanisms. This endeavor enhances our knowledge of disease pathogenesis, contributing to a better understanding of the immune response to *M. tuberculosis*. It provides insight into the specific immune responses to *M. tuberculosis* at the site of infection, which may differ from those in blood. Hence, a study of these T-cell activation pathways in pleural fluid and blood from the same patient with TPE would be able to reveal the role of these events in the dominance of the TH1 profile in TPE. The understanding of human local immune responses to *M. tuberculosis* may facilitate the evaluation of the efficacy of new anti-TB vaccines. Further investigations may unravel the critical targets for therapeutic intervention in chronic inflammatory diseases.

## Data availability statement

The raw data supporting the conclusions of this article will be made available by the authors, without undue reservation.

## Ethics statement

The studies involving human participants were reviewed and approved by National JALMA Institute for Leprosy and Other Mycobacterial Diseases, Agra Human Ethics Committee. The patients/participants provided their written informed consent to participate in this study. Written informed consent was obtained from the individual(s) for the publication of any potentially identifiable images or data included in this article.

## Author contributions

BS conceived, designed the study, performed the experiments, analyzed, interpreted the data, and drafted the manuscript. DR and SU performed the experiments. BJ contributed to designing the study. DC contributed reagents and materials. SK carried out the clinical evaluation of patients with and without tuberculous pleural effusion. BJ, DC, and SK critically reviewed the manuscript. All authors contributed to the article and approved the submitted version.

## Funding

This study is supported by a Department of Biotechnology (DBT) grant under Bio-CARe scheme (Project No. BT/PR18188/BIC/101/884/2016). DR and SU thank the Department of Biotechnology (DBT) for financial support as Project Assistant in the project. The funders had no role in the study design, data collection, analysis, decision to publish, or preparation of the manuscript.

## Conflict of interest

The authors declare that the research was conducted in the absence of any commercial or financial relationships that could be construed as a potential conflict of interest.

## Publisher's note

All claims expressed in this article are solely those of the authors and do not necessarily represent those of their affiliated organizations, or those of the publisher, the editors and the reviewers. Any product that may be evaluated in this article, or claim that may be made by its manufacturer, is not guaranteed or endorsed by the publisher.
